# Fractionated Stereotactic Radiotherapy to Resection Cavities Following Brain Metastasis Surgery: Clinical Outcomes and Challenges

**DOI:** 10.3390/cancers18020304

**Published:** 2026-01-19

**Authors:** Paul Windisch, Robert Förster, Daniel R. Zwahlen, Christina Schröder

**Affiliations:** 1 Department of Radiation Oncology, Cantonal Hospital Winterthur, 8401 Winterthur, Switzerland; 2 Department of Radiation Oncology, Inselspital, Bern University Hospital, University of Bern, 3010 Bern, Switzerland

**Keywords:** brain metastases, postoperative radiotherapy, stereotactic radiotherapy, local control, radiation necrosis

## Abstract

**Purpose:** The purpose of this study was to evaluate local control, survival outcomes, radiation necrosis (RN), patterns of failure, and the role of re-irradiation in patients receiving postoperative fractionated stereotactic radiotherapy (SRT) of brain metastasis (BM) resection cavities. **Methods:** A retrospective, single-centre analysis was conducted on adult patients treated with postoperative RT of BM resection cavities from 2018 to 2024. Each cavity was evaluated individually, including those receiving re-irradiation. MRI-based treatment planning followed institutional SRT standards. Primary endpoints were local control and RN; secondary endpoints included overall survival (OS), failure patterns, and salvage interventions. Kaplan–Meier methods were used for time-to-event analyses. **Results:** Forty-seven patients underwent postoperative RT of 54 cavities, with 10 patients receiving salvage re-irradiation, yielding 67 treatment plans. The median age was 64 years, and non-small cell lung cancer was the most common primary tumour. Fractionation regimens predominantly consisted of 30 Gy in five to six fractions. Median radiologic follow-up was 17 months. Local recurrence, i.e., new or progressive contrast-enhancing tissue within or directly adjacent to the planning target volume, occurred in 12 cavities (22.2%). One- and two-year local control rates were 82.3% and 70.0%. Re-irradiation was used in eight first local failures—most commonly with 30 Gy in six fractions. Two patients required multiple subsequent re-irradiations, and repeat resection was performed in two additional lesions, emphasizing the complexity of managing recurrent disease. Salvage outcomes varied, with some patients achieving durable control after repeated local therapy. RN developed in 18 cavities (33.3%), including 9 cases (16.7%) of grade ≥ 2 RN. RN occurred a median of 15 months after treatment, with high-grade RN presenting earlier (median of 7 months). Four RN events followed re-irradiation, and over half occurred in patients receiving temporally adjacent systemic therapy. Differentiating RN from recurrence remained difficult: in nearly half of cases evaluated with advanced imaging, diagnosis remained uncertain. **Conclusions:** Postoperative fractionated SRT provides moderate long-term local control but is associated with a substantial RN burden, particularly in re-irradiated cavities. Improved imaging, standardized contouring, and optimized treatment strategies are needed to reduce toxicity and enhance outcomes.

## Simple Summary

Brain metastases are common, and surgery is often used to remove them when they cause symptoms. After surgery, focused radiation in the operated area can help prevent the tumour from coming back, but it may also damage healthy brain tissue. This study looked at how effective and safe this type of radiation was in 47 patients treated at one centre. The aim was to see how often tumours returned, how long patients lived, and how often patients developed radiation-related brain injury, which can mimic tumour regrowth on scans and cause symptoms. The results showed that radiation controlled tumour regrowth in most patients. Patients lived a median of about 20 months after treatment. However, one-third of treated areas showed signs of radiation-related brain injury, and in many cases, it was difficult to tell this apart from true tumour return, even with advanced scans. These findings highlight the need for better ways to select patients, plan treatment, and interpret follow-up scans. Improving these areas could reduce harm, avoid unnecessary treatments, and improve quality of life for people living longer with cancer.

## 1. Introduction

Brain metastases (BMs) represent the most common intracranial malignancy in adults, affecting up to 50% of patients with solid tumours [1,2]. Surgical resection remains an important component of management for selected patients, particularly those with symptomatic lesions, mass effect, or diagnostic uncertainty [3]. Postoperative stereotactic radiotherapy (SRT) of the resection cavity has emerged as a critical strategy to improve local control without the neurocognitive drawbacks associated with whole-brain radiotherapy (WBRT), given the potential to deliver highly conformal, dose-intensive therapy while sparing healthy tissue [4,5,6,7,8].

Nevertheless, postoperative SRT of resection cavities raises several challenges. Cavity dynamics are highly variable, with significant shape and volume changes in the early postoperative period [9,10,11]. Therefore, target delineation is variable, and the resulting shapes of the treatment targets may be highly irregular [12]. Moreover, postoperative cavities often exceed the size of intact lesions traditionally treated with SRT, potentially increasing the risk of treatment-related toxicity, particularly radiation necrosis (RN) [13]. Distinguishing RN from tumour recurrence is a persistent diagnostic challenge, even with advanced imaging modalities such as perfusion MRI and 18F-Ethyltyrosin (FET) PET [14,15,16].

This retrospective, single-centre study aims to assess local control, survival outcomes, and RN incidence in patients treated with postoperative fractionated RT of resection cavities following BM resection. The study also evaluates patterns of failure and the use of re-irradiation, providing insight into real-world outcomes in the contemporary era of widespread SRT implementation and improved overall survival due to the availability of efficient systemic therapies for patients with brain metastases.

## 2. Methods

### 2.1. Patient Selection

We conducted a retrospective, single-centre analysis of all patients who underwent postoperative fractionated radiotherapy (RT) of surgical resection cavities after removal of brain metastases between January 2018 and December 2024. This period reflects the widespread implementation of stereotactic radiotherapy (SRT) at our institution. Eligible patients were adults with histologically confirmed solid tumours and at least one brain metastasis treated with surgical resection, followed by local RT of the postoperative cavity. Patients who received whole-brain radiotherapy (WBRT) alone, without focal postoperative treatment, were excluded. For patients with multiple metastases, each resection cavity treated with RT was analysed individually. Patients who underwent re-irradiation of the same cavity due to suspected or confirmed local recurrence were included. Clinical data, imaging reports, surgical notes, radiotherapy plans, and follow-up assessments were extracted from electronic medical records.

### 2.2. Treatment Planning and Delivery

MRI planning typically took place within one week before the start of SRT. The GTV was contoured as the entire postoperative cavity and any residual disease (if applicable) and was delineated according to MRI findings (T1 contrast). In cases of re-irradiation of a localized recurrence within a formerly treated cavity, only the area of recurrence was targeted, rather than re-treating the full cavity. In cases of multiple recurring lesions within the cavity or after re-resection, re-irradiation of the entire cavity was also permitted. A CTV expansion of 3–5 mm along the dura was permitted, as per consensus guidelines [12], followed by a uniform 2 mm PTV margin. Patients were immobilized using thermoplastic masks. All treatments were performed using the strict IGRT protocol. Treatment planning was performed using Eclipse (Varian Medical Systems, Palo Alto, CA, USA), and treatments were delivered on C-arm linear accelerators (Varian TrueBeam, Varian Medical Systems, Palo Alto, CA, USA). Patients were treated either on consecutive or alternating days.

### 2.3. Endpoints

Endpoints were local control and the incidence of radiation necrosis (RN). Local recurrence was defined radiographically as new or progressive contrast-enhancing tissue within or directly adjacent to the planning target volume, assessed by board-certified neuroradiologists. RN was diagnosed based on a combination of clinical presentation and radiographic findings suggestive of treatment-related injury; when available, histopathology from repeat resection was also considered. When differentiation between RN and local failure was challenging, the diagnosis considered most probable was chosen according to the later progression of the lesion. Secondary endpoints included overall survival (OS); patterns of failure; and rates of re-treatment, including re-irradiation, repeat surgical resection, and salvage WBRT. Time-to-event endpoints were calculated from the date of the first postoperative RT. Advanced imaging modalities including functional MRI and FET PET were documented when used to distinguish RN from recurrent tumours.

### 2.4. Statistical Analysis

Descriptive statistics were used to summarize patient demographics, tumour characteristics, treatment parameters, and follow-up outcomes. Continuous variables are reported as medians with ranges, and categorical variables are reported as frequencies and percentages. OS, local control, and time to RN were estimated using Kaplan–Meier methods. Patients were censored at the last radiographic follow-up or death as appropriate. The incidence of RN was analysed on a per cavity basis, and separate analyses were performed for all RN and grade ≥2 RN (moderate symptoms with corticosteroids indicated according to the Common Terminology Criteria for Adverse Events (CTCAE version 5) scoring system). No formal multivariate modelling was conducted due to sample-size limitations. All statistical analyses were performed using SPSS Version 30 (IBM SPSS Statistics). Visualizations were created using Python (v. 3.13.2) and the seaborn (v. 0.13.2) package.

## 3. Results

### 3.1. Patient and Treatment Characteristics

A total of 280 patients were treated with cranial stereotactic radiotherapy in the abovementioned interval. Of those, 47 patients (53% male and 47% female) underwent postoperative fractionated RT of a total of 54 resection cavities. A total of 10 patients required fractionated re-irradiation to resection cavities for local recurrence, resulting in 67 total treatment plans. Median age at initial RT was 64 years (range, 43–77 years). Non-small cell lung cancer (NSCLC) was the most frequent primary tumour (38.3%), followed by breast cancer (12.8%) and lower gastrointestinal tumours (colon and sigma carcinoma, 8.6%).

A total of 19 patients (40.4%) did not receive systemic therapy during or after SRT of the resection cavities, 4 of whom received no systemic therapy at all during their entire course of disease. A total of 16 patients (34.0%) received systemic therapy within 1 month after SRT. The majority of those patients received immunotherapy (six patients), a combination of chemotherapy and immunotherapy (four patients), or chemotherapy alone (three patients).

**Figure 1:**
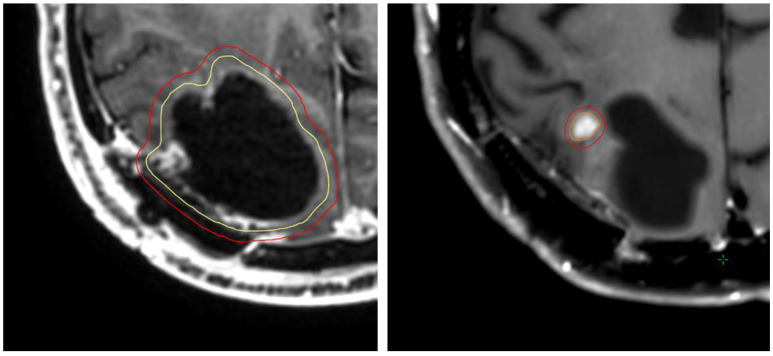
Patient example. (**Left**) Initial target of the cavity; (**right**) target of the localized re-irradiation of the small local recurrence.

Fractionation schedules reflected contemporary SRT practice. The most frequently used regimens were 30 Gy in five fractions (80% Isodose line (IDL), 26.9%) and 30 Gy in six fractions (80% IDL, 47.8%). No radiosurgery procedure was applied in this cohort. In-field re-irradiation in cases of recurrence was performed in eight patients; one patient underwent two re-irradiations, and one underwent three. Figure 1 shows a patient example.

Further patient and treatment-related characteristics are shown in Table 1 and Table 2.

### 3.2. Local Control and Treatment of Local Recurrence

**Figure 2:**
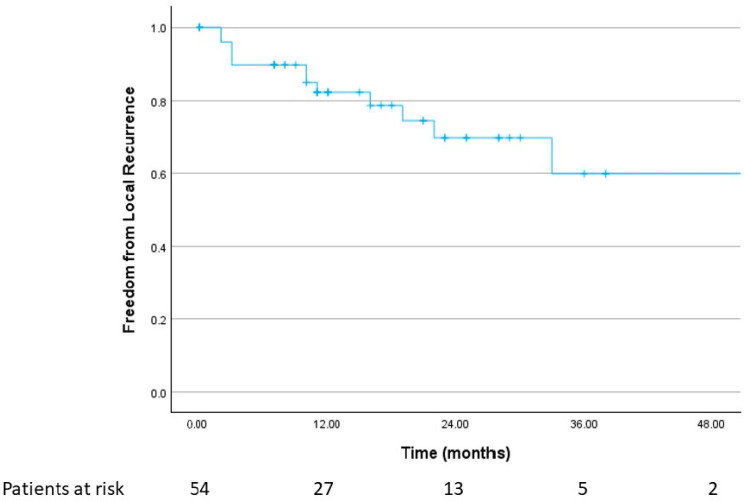
Freedom from local recurrence after first SRT (*n* = 54).

Median radiological follow-up was 17 months (range, 1–79 months), with 28 patients followed for ≥ 12 months. Local failure after first radiotherapy occurred in 12 of 54 cavities (22.2%). The 1- and 2-year local control rates were 82.3% and 70%. If a local failure occurred, the median time to failure was 10 months (range, 2–33 months). Figure 2 shows the respective Kaplan–Meier curves for freedom from local recurrence.

Among cavities with local recurrence, for the first recurrence, eight lesions (14.8%) underwent re-irradiation with local SRT. In cases of re-irradiation, the most common fractionation was 30 Gy in 6 fractions (80% IDL, 3 lesions), followed by 30 Gy in 5 fractions (80% IDL, 2 lesions) and 30 Gy in 10 fractions (80% IDL, 2 lesions). In three lesions, re-irradiation was applied to the macroscopic recurrence only, while in six lesions, re-irradiation was applied to the entire cavity. In two lesions, there was another in-field failure after re-irradiation. One lesion recurred 9.7 months after re-irradiation and was treated with another course of irradiation of the macroscopic lesion only (30 Gy in six fractions, 80% IDL). Another lesion had a further in-field recurrence after 43.6 months and was treated with another course of SRT of the recurrence only (30 Gy in six fractions, 80% IDL). This patient went on to have a third in-field recurrence after 14.5 months, which was, again, treated with SRT of the recurrence only (30 Gy in five fractions, 80% IDL).

Two lesions were treated with repeat resection followed by additional irradiation. Time to in-field recurrence from first SRT for those lesions was 2.7 and 4.0 months, respectively. Both were re-irradiated with 30 Gy in 10 fractions and 35 Gy in 10 fractions (both 80% IDL) of the whole cavity. One of those lesions had another in-field failure 35.9 months after re-treatment, which was treated with another SRT of the macroscopic recurrence only.

One patient with in-field failure of two resection cavities received WBRT due to concurrent out-of-field intracranial failure.

### 3.3. Overall Survival

**Figure 3:**
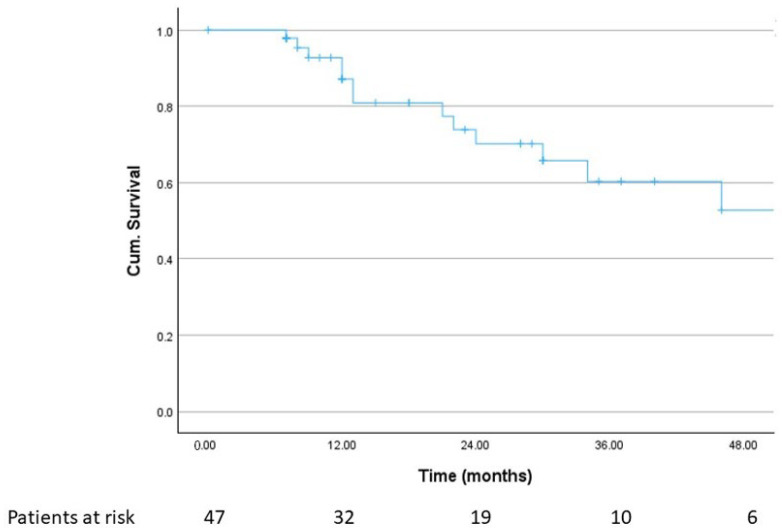
Overall survival (*n* = 47).

Median OS for all patients from the first SRT of a resection cavity was 64 months (95% CI, 24.1–103.9 months), and the 1- and 2-year OS rates were 87.1% and 70.2%, respectively. Figure 3 shows the corresponding Kaplan–Meier curve.

When looking at patients with NSCLC separately, the median OS was not reached (95% CI, 2.1 months—not reached), with 1- and 2-year OS of 92.3% and 79.1%. For non-NSCLC patients, the median OS was 46.5 months (95% CI, 21.8 months—not reached), with 1- and 2-year OS of 92.5% and 70.1%, respectively.

### 3.4. Radiation Necrosis

RN developed in 18 cavities (33.3%). Four cases occurred after re-irradiation, and nine cases (16.7% of all cavities) met criteria for grade ≥ 2 RN. Median time to RN was 15 months (range, 2–79 months). Grade ≥ 2 RN occurred earlier, with a median onset of 7 months.

Of the 18 RN cases, 4 (22.2%) occurred after re-irradiation. In 10 RN cases (55.6%), the respective patients were treated with concurrent or adjuvant systemic therapy.

**Figure 4:**
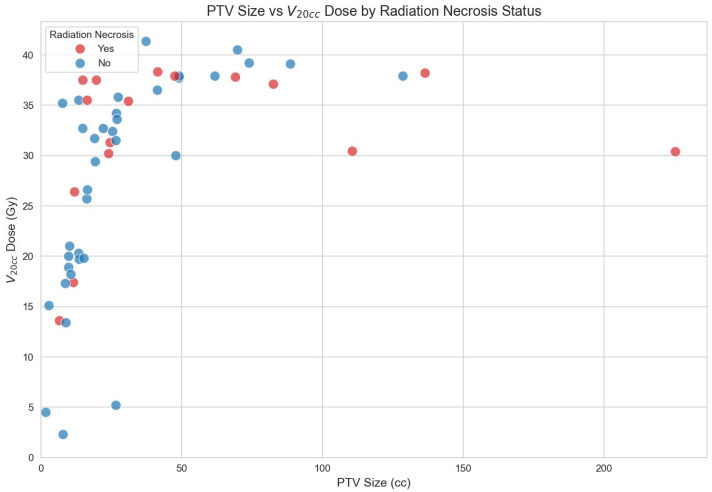
PTV size and V_20cc_ of the brain in patients with and without RN.

In patients without in-field re-irradiation, the median size of the PTV in patients without RN was 19.27 cc, while that in patients with RN was 27.89 cc. This was not statistically significant, though. Figure 4 shows a scatter plot of target size, the V_20cc_ dose of the normal brain tissue, and whether or not patients developed RN after the first RT.

The distinction between RN and local recurrence remained challenging despite advanced imaging. Functional MRI or FET-PET was used in 14 cases, yet diagnosis remained difficult in 8 lesions. In these cases, the further course during follow-up was used to classify lesions as suspected RN or suspected true progression.

## 4. Discussion

This retrospective analysis highlights the complexities of delivering SRT to resection cavities in patients with BMs. Local control rates in our cohort are consistent with previously published data, supporting the effectiveness of fractionated cavity stereotactic radiotherapy (SRT) [6,17,18]. Nevertheless, long-term outcomes indicate that there remains room for improvement. The observed local recurrence rate of 22.2% reflects the inherent challenges of treating large or irregular postoperative cavities, where achieving sufficient target coverage while minimizing toxicity requires a careful balance.

The incidence of RN in this cohort was notable, with one-third of cavities affected and grade ≥ 2 RN observed in 16.7%. The reported rates in the literature vary due to the diagnostic uncertainty, definition and grade of reported RN, and follow-up of the cohorts. According to the meta-analysis by Akanda et al., most studies report an incidence rate below 10%, although some studies report higher rates similar to the rate in this cohort [18]. Such rates may reflect both the relatively large cavity sizes treated in our cohort and the longer survival observed in contemporary patients (median overall survival in this cohort was 64 months). Importantly, RN may be underdiagnosed or misclassified due to the frequent difficulty in distinguishing radiation-induced injuries from tumour recurrence. Even with advanced imaging modalities like FET, PET, MRI, and functional MRI, nearly half of the evaluated cases remained inconclusive. This diagnostic uncertainty has major consequences for patient management, often prompting re-irradiation or surgery in cases where the underlying pathology remains unclear [14,15,16]. Better differentiation of local recurrence and radiation-induced changes remains a crucial topic of research, especially in the era of modern systemic therapies and longer patient survival.

One important aspect potentially influencing both local control and toxicity is the choice of fractionation. In contrast to single-fraction stereotactic radiosurgery (SRS), fractionated SRT allows for delivery of the total dose over multiple treatment sessions, thereby reducing the dose per fraction to surrounding normal brain tissue. This approach is particularly relevant in the postoperative setting, where resection cavities are often larger than intact metastases and may be adjacent to critical structures.

Several studies and a meta-analysis have demonstrated that fractionated regimens provide better 12-month local control to single-fraction SRS. In the meta-analysis by Akanda et al., the 12-month local control after SRS was found to be 80%, compared to 87.3% after fractionated treatment, which is statistically significant [18].

The predominance of 30 Gy in five or six fractions in our cohort reflects contemporary practice patterns aimed at balancing tumour control with safety. Still, the optimal fractionation remains unknown.

Another factor to consider is that fractionation, itself, introduces additional uncertainties. Postoperative cavities are dynamic structures that can change in size and shape over time, particularly in the early weeks following surgery [9,10,11]. Therefore, longer fractionation schedules may be more susceptible to anatomical changes during treatment, potentially leading to geographic misses or unnecessary irradiation of normal brain tissue. This issue underscores the importance of high-quality image guidance and careful target delineation, as well as consideration of adaptive strategies in selected cases. While ultra-hypofractionated regimens (e.g., three fractions) may reduce the overall treatment time and limit the window for cavity evolution, they deliver a higher dose per fraction and may increase the risk of RN, particularly in large cavities.

These findings also raise important questions regarding the necessity of routine, upfront postoperative SRT in all patients with brain metastases, particularly those who achieve a gross total resection (GTR). In the original phase 3 study by Mahajan et al., the 12-month freedom from local recurrence was significantly better in the SRS group (72% versus 43% in the observation group) [6]. However, there was no statistically significant difference for either OS or freedom from distant brain recurrence.

Especially in selected patients like those receiving modern systemic therapies with high intracranial response rates, initial observation with close imaging surveillance may be a reasonable alternative to upfront radiotherapy. The data for intact brain metastases for many primary entities show excellent cranial control rates [19,20,21,22,23]. Such an approach could spare patients from potentially morbid local therapy while preserving the option for salvage treatment should a true recurrence occur.

Re-irradiation was used frequently in our cohort, demonstrating its perceived value as a salvage strategy. However, data on re-irradiation of resection cavities is rare. Re-irradiation likely contributed to increased RN risk in these patients, emphasizing the need to optimize initial treatment plans and follow-up strategies to minimize the need for retreatment [24]. As systemic therapies continue to extend survival, the cumulative effects of intracranial treatments, including RN, will become increasingly significant.

Overall, the results underscore the need for improved postoperative SRT planning approaches, including better identification of patients at high risk for toxicity. In this context, preoperative SRS or SRT represents a promising alternative to postoperative treatment, with the potential advantages of improved target definition, smaller irradiated volumes, and possibly lower rates of RN [13,25,26,27,28,29]. While postoperative SRT remains the current standard in many centres, accumulating evidence suggests that preoperative approaches may offer meaningful benefits for selected patients. Prospective randomized trials comparing preoperative and postoperative SRT are recruiting and will help to define optimal patient selection, fractionation strategies, and long-term outcomes and will ultimately guide the integration of preoperative SRT into routine clinical practice [30].

The limitations of this study include its retrospective design, the small sample size, and the fact that all patients were treated with fractionated SRT, which may limit the generalizability of the findings. Despite these limitations, the data provide valuable insights into the evolving role of postoperative cavity irradiation in the modern treatment landscape of brain metastases.

## 5. Conclusions

In this single-centre, retrospective cohort, postoperative fractionated RT of resection cavities after BM resection yielded moderate local recurrence rates and a substantial incidence of high-grade radiation necrosis, particularly among patients with extended follow-up and after re-irradiation. These findings highlight persistent challenges in the management of postoperative cavity irradiation, including the complexity of target delineation, evolving anatomy, and limitations in differentiating RN from tumour recurrence. As patient survival improves, optimizing treatment strategies and developing reliable imaging tools will be essential to reduce toxicity and enhance long-term outcomes. Further prospective studies and further technological innovation are warranted to refine postoperative SRT and improve patient care.

## Figures and Tables

**Table 1:** Patient and systemic treatment-related characteristics (*n* = 47).

	*n*	%
**Sex**		
F	22	46.8
M	25	53.2
**Entity**		
NSCLC AdenoCa	13	27.7
NSCLC SCC	3	6.4
NSCLC NOS	2	4.3
Breast Cancer	6	12.8
SCLC	3	6.4
Colon/SigmaCa	4	8.6
Melanoma	2	4.3
others	14	29.8
**Systemic therapy during/after first SRT**		
None	19	40.4
1	22	46.8
2	3	6.4
3	3	6.4
**First line during/after SRT**		
Targeted Therapy + Chemotherapy	4	18.2
Targeted Therapy only	4	18.2
Immunotherapy + Chemotherapy	6	27.3
Immunotherapy only	6	27.3
Chemotherapy	7	31.8
unknown	1	4.5
Total	47	100

**Table 2:** Radiotherapy-related characteristics at first SRT (*n* = 54).

	*n*	%
SRT fractionation		
3x9@80% IDL (alternating days)	1	1.9
3x9@80% IDL (consecutive days)	1	1.9
5x6@80% IDL (alternating days)	11	20.4
5x6@80% IDL (consecutive days)	5	9.3
6x5@80% IDL (alternating days)	8	14.8
6x5@80% IDL (consecutive days)	19	35.2
others	9	16.7
Total	54	100
GTV size (cc) median (range)		
1st course	13.3 (0.49–140.08)	
2nd course	5.73 (0.3–146.31)	
3rd–4th course	0.88 (0.3–5.7)	
PTV size median (range)		
1st course	23.17 (1.83–225.37)	
2nd course	14.2 (1.04–251.31)	
3rd–4th course	2.27 (0.7–8.61)	

## Data Availability

No primary data is available due to ethical restrictions.
